# Sustained eradication of hepatitis C virus by low-dose long-term interferon therapy in a renal transplant recipient with dual infection with hepatitis B and C viruses: a case report

**DOI:** 10.1186/1752-1947-5-246

**Published:** 2011-06-29

**Authors:** Ming-Ling Chang, Ping-Chin Lai, Chau-Ting Yeh

**Affiliations:** 1 Liver Cancer Research Center, Chang Gung Memorial Hospital, 6J Laboratory, Linko Medical Center, 199 Tung Hwa North Road, Taipei, Taiwan; Graduate Institute of Clinical Medical Sciences, Chang Gung University, College of Medicine, Taoyuan, Taiwan; 2 Department of Nephrology, Chang Gung Memorial Hospital, Linko Medical Center, 199 Tung Hwa North Road, Taipei, Taiwan; 3 Liver Cancer Research Center, Chang Gung Memorial Hospital, 6J Laboratory, Linko Medical Center, 199 Tung Hwa North Road, Taipei, Taiwan; Graduate Institute of Clinical Medical Sciences, Chang Gung University, College of Medicine, Taoyuan, Taiwan

## Abstract

**Introduction:**

Accelerated liver function deterioration has been recognized in renal transplant recipients infected with hepatitis C virus (HCV). Although combination therapy with interferon plus ribavirin has been established as the standard treatment for patients with chronic HCV, the high risk of allograft rejection associated with interferon therapy has greatly discouraged the clinical use of this regimen. In Asia, where chronic hepatitis B virus (HBV) is prevalent, dual infection with HBV and HCV poses an even greater challenge for clinical hepatologists.

**Case presentation:**

In this article, we report the case of a 51-year-old Taiwanese man with dual infection with HBV and HCV prior to renal transplantation. Low-dose interferon (3 to 6 × 10^6 ^U/week) and ribavirin (100 mg/day to 200 mg/day) were prescribed following the reactivation of the man's HCV after renal transplantation. Additionally, lamivudine (100 mg/day) was administered concomitantly to prevent HBV reactivation. His initial serum HCV RNA concentration was 5.2 × 10^6 ^copies/mL (genotype 2a). After three and one-half years of antiviral therapy, his HCV was successfully eradicated without any episodes of allograft rejection. His serum HCV RNA remained negative six months after withdrawal from interferon and ribavirin treatment. His serum HBV DNA remained undetectable throughout the course of therapy.

**Conclusion:**

Low-dose, long-term interferon therapy may achieve sustained eradication of HCV in the renal transplant recipient with dual infection with HBV and HCV.

## Introduction

Chronic hepatitis C virus (HCV) infection may lead to severe sequels such as liver cirrhosis and hepatoma [1]. It was recognized as an important cause of morbidity and mortality in renal transplant recipients [2]. Besides accelerated deterioration of liver function in chronic HCV infection, HCV-related glomerulopathy [3] and HCV-associated fibrosing cholestatic hepatitis [4] have also been documented in renal transplant recipients. Although interferon plus ribavirin combination therapy has been established as the standard treatment for chronic HCV infection, the risk of acute rejection associated with interferon administration has greatly hindered the use of this treatment [5]. In an area where chronic hepatitis B virus (HBV) infection is prevalent, such as Asia, the challenge is even greater, because dual infection with HBV and HCV may be encountered. Mutual interference of HCV and HBV replication has been reported in many studies with alternately dominant hepatitis activities caused by HBV and HCV [6]. More severe liver diseases have been observed in patients with dual HBV and HCV infections. The strategy for treating a renal transplant recipient carrying both HBV and HCV with deteriorating hepatic function remains undetermined. In this article, we report the case of a renal transplant recipient who had dual infection with HBV and HCV prior to undergoing transplantation. Low-dose interferon and ribavirin were prescribed following the reactivation of HCV infection in this patient due to the use of immunosuppressants. The patient's serum HCV RNA was successfully eradicated after three and one-half years of antiviral therapy without any episodes of allograft rejection.

## Case presentation

A 51-year-old Taiwanese man had received regular hemodialysis three times weekly for six years when he was diagnosed with end-stage renal disease. Chronic HBV and HCV infection had been diagnosed at the beginning of hemodialysis on the basis of serological tests. His HBV surface antigen (HBsAg) test had been positive since his first hospital visit, and his antibody against HCV was positive three years after the initiation of hemodialysis. His HBV DNA was assayed twice seven years later. The results of his HCV-RNA and HBV-DNA quantitative tests were 5.9 × 10^5 ^copies/mL and 4.1 × 10^6 ^copies/mL, respectively. Cadaveric renal transplantation was performed six years after his hemodialysis treatment was initiated. Immunosuppressants had been administered since that time, including prednisolone, mycophenolate mofetil, cyclosporine and tacrolimus. Over the next two years, he was admitted to our hospital 14 times for various reasons, including pulmonary cytomegalovirus infection, urinary fungal infection, lip herpes viral infection, acute allograft rejection, pulmonary tuberculosis, tuberculous cystitis, hyperuricemia and ureter stricture with obstructive nephropathy. One year after he underwent renal transplantation, antituberculosis therapy was administered for one year with treatment regimens of isoniazid, rifampin, and ethambutol for three months, followed by isoniazid alone for seven months.

**Figure 1:**
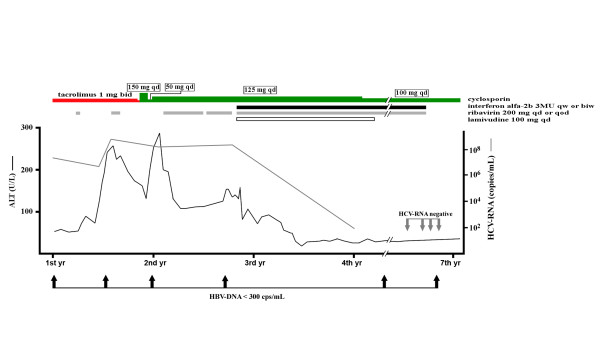
**Clinical course of a renal transplant recipient infected with hepatitis B and C viruses (HBV and HCV, respectively)**. 1st yr~7th yr, first year to seventh year of main treatment; gray lines and arrows, HCV RNA levels; solid lines and arrows, HBV DNA levels; red bar, tacrolimus; green bar, cyclosporine; solid bar, interferon α-2b; gray bar, ribavirin; empty bar, lamivudine.

His liver biochemistry tests (aspartate aminotransferase, alanine aminotransferase (ALT) and bilirubin) were normal until three years after renal transplantation, when mild elevated aminotransferase levels about one to threefold the upper limit of normal were noted. Prednisolone was withdrawn at the same time. Marked elevation of aminotransferase levels (up to sevenfold the upper limit of normal) associated with jaundice (bilirubin 3 mg/dL to 4 mg/dL) were found one year after the administration of prednisolone. His HBV DNA was below the detection limit (<300 copies/mL), whereas his HCV RNA was 5.2 × 10^6 ^copies/mL. A HCV genotype assay indicated genotype 2a. One year later lamivudine therapy (100 mg/day) was prescribed and was continued for the next two years. Upon the acute exacerbation of liver function, ribavirin monotherapy was given intermittently at 200 mg/day for nine months and 200 mg every other day for the following two months. A liver biopsy was taken one year later, which revealed chronic active hepatitis with a Knodell Histology Activity Index score of 10 (periportal necrosis, 3; intralobular necrosis, 1; portal inflammation, 3; and fibrosis, 3) as well as marked fatty change. The patient's immunohistochemistry study for HBsAg was positive. Combination therapy of ribavirin (200 mg/day or every other day) and interferon α-2b (3 × 10^6 ^U by subcutaneous injection once to twice weekly) was started one year after his acute exacerbation of liver function. The doses were adjusted according to the tolerability of the host as well as the blood tests (Figure 1). His combination therapy was ended in four years. His ALT levels fluctuated between 100 U/L and 260 U/L (up to sevenfold the upper limit of normal) during the first two years of his acute liver exacerbation, which decreased gradually thereafter. Ultimately, his ALT levels remained between one- and twofold the upper limit of normal. Sequential follow-up with liver sonography showed mild parenchymal liver fibrosis with moderate change in fatty liver. Negative results of his serum HCV RNA were documented once one year before the end of combination therapy and three times at the end of the combination therapy. His HBV DNA was below the detection limit after the withdrawal of prednisolone therapy (a total of six times over the next six years).

## Discussion

Coinfection of HBV and HCV is a complicated issue commonly encountered in an area where HBV is prevalent, such as Asia. Dual HBV and HCV infections have been found to accelerate the clinical deterioration of patients with liver disease compared with patients with a single hepatitis virus infection [6]. In patients undergoing organ transplantation, liver diseases are even more difficult to control, owing to the need for extensive immune suppression. In patients undergoing liver transplantation, long-term lamivudine therapy has been recommended for HBV carriers to prevent reactivation of the disease [7]. However, in renal transplant recipients, the beneficial effect is not well-established. Mutual interference of viral replication between HBV and HCV has been described in several reports [5,6]. In our patient, HBV DNA was initially detectable (5.9 × 10^5 ^copies/mL to 4.1 × 10^6 ^copies/mL), but became negative during flares of HCV. It is possible that after HCV was suppressed by antiviral therapy, HBV could have been reactivated. Lamivudine was thus given concomitantly for this patient. In our hospital, lamivudine was not available until 1999, and, under the National Health Insurance Policy in Taiwan, the duration of lamivudine use was limited to one and one-half years for each patient.

Although combination therapy with interferon plus ribavirin has been established as the standard treatment for chronic HCV infection, the strategy for posttransplantation anti-HCV therapy remains inconclusive [2]. In our patient, a liver biopsy prior to anti-HCV therapy revealed obvious fibrosis and necroinflammation. If these conditions had been left untreated, the chance that this patient would develop liver cirrhosis was great. They are also the main cause of hepatocellular carcinoma [8]. The HCV RNA genotype in this patient was 2a, a favorable factor for anti-HCV therapy [9,10]. Attempts have been made to treat HCV infection in renal transplant recipients using various regimens, including ribavirin alone, combination therapy with ribavirin and amantadine [10], interferon in combination with ribavirin [5,11] and interferon alone [12]. Combination therapy of ribavirin with amantadine was found not to be superior to ribavirin monotherapy, which resulted in a good biochemical response but was not associated with virological clearance [5]. Intravenous interferon-β therapy given daily for six weeks was reported to induce seroclearance of HCV in a renal transplant recipient with stable renal function [12]. Low-dose interferon-α in combination with ribavirin was effective in some patients after a minimum therapeutic period of six months, although it was poorly tolerated and resulted in graft dysfunction in a significant number of patients [5]. Ultra-low-dose interferon-α (1 × 10^6 ^U given subcutaneously three times weekly) plus ribavirin (600 mg/day) for 48 weeks was reported to clear HCV RNA in five of 11 renal transplant recipients, but acute graft failure (in one patient) and sepsis (in two patients) also occurred [11]. Taken together, interferon and ribavirin could eradicate HCV infection in selected renal transplant recipients, but the dose needs to be adjusted as needed to avoid allograft rejection. On the other hand, recent studies have indicated that patients with chronic HCV who fail to achieve viral clearance could benefit from long-term low-dose interferon maintenance therapy and that the histological deterioration in these patients could be prevented [13,14]. In the present case, the course of HCV therapy lasted for three and one-half years, which is much longer than any other case reported in the literature. Neither allograft rejection nor significant infection was noted. Intriguingly, HCV infection was successfully eradicated at the end of therapy and no relapse has occurred to date. Interferon-α rather than pegylated interferon was chosen as the therapy for this patient because the longer half-life of the latter regimen might be associated with a higher chance of allograft rejection. After the end of treatment, the patient's ALT levels were still mildly elevated. Since both his HBV DNA and HCV RNA were negative, it was likely that fatty liver rather than viral hepatitis accounted for his elevated aminotransferase levels.

## Conclusions

Low-dose long-term interferon therapy could achieve sustained eradication of HCV infection in renal transplant recipients with dual HBV and HCV infections.

## Consent

Written informed consent was obtained from the patient for publication of this case report and any accompanying images. A copy of the written consent is available for review by the Editor-in-Chief of this journal.

## Competing interests

The authors declare that they have no competing interests.

## Authors' contributions

CTY analyzed and interpreted the patient data regarding his liver disease. PCL analyzed and interpreted the patient data regarding the patient's renal disease. MLC was a major contributor to the writing of the manuscript and analyzed all the data. All authors read and approved the final manuscript.
